# The Hippo pathway in tissue homeostasis and regeneration

**DOI:** 10.1007/s13238-017-0371-0

**Published:** 2017-01-27

**Authors:** Yu Wang, Aijuan Yu, Fa-Xing Yu

**Affiliations:** 1 Children’s Hospital and Institutes of Biomedical Sciences, Fudan University, Shanghai, 200032 China; 2 Collaborative Innovation Center of Genetics and Development, School of Life Sciences, Fudan University, Shanghai, 200433 China; 3 Key Laboratory of Reproduction Regulation of NPFPC, SIPPR, IRD, Fudan University, Shanghai, 200032 China

**Keywords:** Hippo, YAP, regeneration

## Abstract

While several organs in mammals retain partial regenerative capability following tissue damage, the underlying mechanisms remain unclear. Recently, the Hippo signaling pathway, better known for its function in organ size control, has been shown to play a pivotal role in regulating tissue homeostasis and regeneration. Upon tissue injury, the activity of YAP, the major effector of the Hippo pathway, is transiently induced, which in turn promotes expansion of tissue-resident progenitors and facilitates tissue regeneration. In this review, with a general focus on the Hippo pathway, we will discuss its major components, functions in stem cell biology, involvement in tissue regeneration in different organs, and potential strategies for developing Hippo pathway-targeted regenerative medicines.

## Introduction

Tissue damage, such as traumatic or surgical injury, infection, or aging, results in the loss of cells and tissue. To maintain their physiological functionality and morphology, damaged organs must be repaired or regenerated. Some lower organisms, such as planarian and salamanders, can effectively regenerate following injury. However, most mammals have limited regenerative potential, and only a few organs, such as the liver, skin, and intestine, have some regenerative capability (Whyte et al., 2012). In some organs, such as the skin, the limited regenerative capability is compensated by excess fibrosis, which results in tissue scaring (Gurtner et al., 2012).

Tissue regeneration is a complex process involving multiple cell types. First, the tissues surrounding the damaged sites need to induce cell proliferation and differentiation to supply necessary tissue-specific cells acting as building blocks for regeneration. Second, the vascular, nervous, and immune systems as well as the extracellular matrix (ECM) need to be restored to maintain functionality of the new tissue. Thus, different cell populations are required to work in a cooperative manner to support a successful tissue regeneration (Carlson, 2007).

The origin of these “new” cells during regeneration remains controversial. At least four different mechanisms have been suggested: 1) proliferation of terminally differentiated cells (usually polyploid cells); 2) dedifferentiation of mature cells; 3) expansion and differentiation of resident progenitor cells; and 4) influx of stem cells from other tissues. It is likely that different organs employ a unique mechanism in a tissue-specific manner (Carlson, 2007).

Tissue regeneration also involves diverse cellular signaling pathways (Stoick-Cooper et al., 2007). For example, Wnt signaling plays a vital role in intestinal regeneration (Barker, 2014), hepatocyte growth factor (HGF) signaling is required for liver regeneration (Borowiak and Wigler, 2004; Huh et al., 2004), and bone morphogenetic protein (BMP) signaling is critical in digit tip regeneration (Han et al., 2003). Following injury, multiple signaling pathways are coordinated spatiotemporally, in a tissue and context-dependent manner, to ensure a successful regeneration program.

The Hippo pathway is a relatively new signaling pathway involved in tissue homeostasis, organ size control, and tumorigenesis (Yu et al., 2015b). Here, we will review what is currently known about the role of the Hippo pathway in modulating tissue regeneration.

## The Hippo Pathway

**Figure 1:**
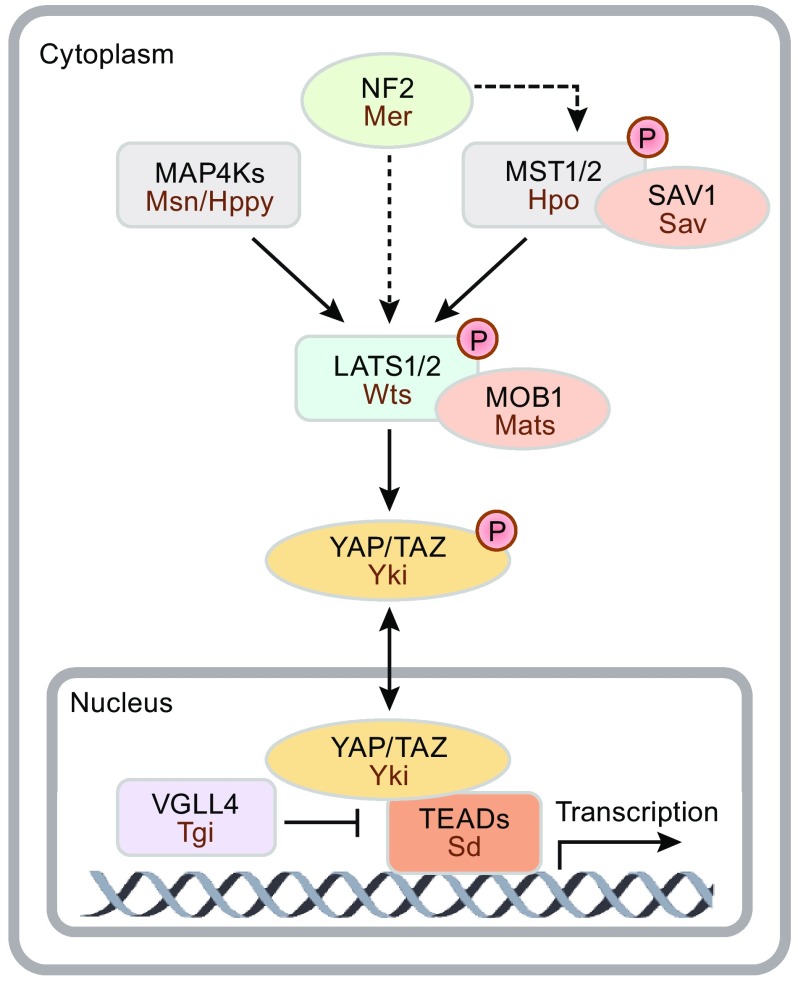
**The Hippo signaling pathway**. Major mammalian Hippo signaling pathway components and their *Drosophila* orthologues are also shown. Abbreviations: Yes Associated Protein (YAP), Transcriptional Co-Activator With PDZ-Binding Motif (TAZ, also known as WWTR1), TEA Domain Transcription Factor (TEAD), Vestigial Like Family Member 4 (VGLL4), Large Tumor Suppressor Kinase 1/2 (LATS1/2), Mammalian STE20-Like Protein Kinase 1/2 (MST1/2, also known as STK4/3), MOB Kinase Activator 1 (MOB1), Salvador (SAV1), Mitogen-Activated Protein Kinase Kinase Kinase Kinase (MAP4K), Neurofibromin 2 (NF2, also known as Merlin), Yorkie (Yki), Hippo (Hpo), Warts (Wts), Merlin (Mer), Misshapen (Msn), Happyhour (Hppy), Salvador (Sav), Marts (Mats), Scalloped (Sd), Tondu-domain-containing Growth Inhibitor (Tgi)

The Hippo pathway has been established in *Drosophila melanogaster* as an important regulator of organ size, and this pathway is highly conserved in mammals (Pan, 2010; Halder and Johnson, 2011; Yu and Guan, 2013). The core Hippo pathway consists of a kinase cascade (Fig. 1). MST1/2 and MAP4Ks phosphorylate LATS1/2, leading to LATS1/2 activation (Full names of Hippo pathway components are shown in legends of Fig. 1). Activated LATS1/2 then phosphorylate YAP/TAZ, which results in YAP/TAZ inactivation. As the major downstream effectors of the Hippo pathway, unphosphorylated YAP/TAZ translocate to the nucleus and induce target gene transcription by interacting with the transcription factors TEADs (TEAD1–4). In addition, SAV1 and MOB1 are scaffold proteins for MST1/2 and LATS1/2, respectively, and upstream regulators such as NF2 can also induce LATS1/2 activity. Furthermore, the interaction between YAP/TAZ and TEADs is antagonized by VGLL4. The *Drosophila* orthologues of these Hippo pathway components are also shown in Fig. 1.

The Hippo pathway is regulated by a variety of signals including cell polarity, cell-cell contact, cell-ECM interaction, mechanical cues, and diffusible signals including a variety of G-protein-coupled receptor (GPCR) ligands (Yu et al., 2015b). These upstream signals of the Hippo pathway are important constituents of the stem cell niche, and undergo dynamic changes upon tissue injury. Thus, in response to injury-derived signals, the Hippo pathway may function as an immediate mechanism to mobilize tissue resident progenitor cells and initiate tissue regeneration.

## The Hippo Pathway in Stem Cell Biology

The proliferation, differentiation, and migration of stem cells are crucial during tissue regeneration, and the Hippo pathway has been shown as an important regulator in stem cell function. The first cell lineage specification during embryonic development is the emergence of the inner cell mass (ICM) and trophectoderm (TE), and the Hippo pathway plays an essential role in this process (Sasaki, 2015). High YAP activity is required for TE specification, and in mice, the cell fate of trophoblasts (TE) and embryoblasts (ICM) can be interconverted by manipulating *Yap*/*Taz* or their upstream regulators (Cockburn et al., 2013; Hirate et al., 2013; Lorthongpanich et al., 2013). This suggests that the Hippo pathway plays a key role in regulating stem cell biology at early embryonic stage.

The function of the Hippo pathway has been well studied in both embryonic stem cells (ESCs) and induced pluripotent stem cells (iPSCs). YAP is highly expressed in self-renewing ESCs but is inactivated during differentiation (Lian et al., 2010; Tamm et al., 2011). YAP may induce the expression of pluripotency-associated genes which promote ESC self-renewal. Overexpression of *Yap* inhibits ESC differentiation and maintains stem-like properties and self-renewal even under differentiation conditions, while *Yap*/*Taz* knockdown is sufficient to result in the loss of the ESC phenotype (Varelas et al., 2008; Lian et al., 2010; Tamm et al., 2011; Beyer et al., 2013). Likewise, knockdown of *Lats2* increases the reprograming efficiency of iPSCs (Qin et al., 2012). Deletion of *Mst1*/*2* in ESCs causes enhanced cell proliferation and impaired differentiation (Li et al., 2013). Moreover, stem cells overexpressing *YAP* reveal naïve state-like properties (identical to stem cells from pre-implantation embryos), and the YAP activator lysophosphatidic acid (LPA) can partially substitute for YAP to promote the transition to naïve state (Qin et al., 2016).

Several recent studies suggest that YAP is dispensable for self-renewal but required for differentiation of ESCs. Knockdown or knockout of *Yap* does not alter ESC self-renewal but impairs their differentiation (Azzolin et al., 2014; Chung et al., 2016). TAZ and the TEADs are also dispensable for ESC self-renewal (Chung et al., 2016). In addition, deletion of *Lats2* in ESCs impairs both their pluripotency and ability to differentiate (Aylon et al., 2014). This discrepancy between these studies is likely due to the high sensitivity of the Hippo pathway to different cell culture conditions. Indeed, most experiments on pluripotent stem cells are performed on cultured cells, and experimental settings may differ. Thus, further investigations are required to gain a better understanding of the function of YAP/TAZ in PSCs. Nevertheless, the mechanisms regulating the Hippo pathway in PSCs may be shared by tissue-resident progenitor cells involved in tissue regeneration.

## The Hippo Pathway in Mammalian Tissue Regeneration

The Hippo pathway has been shown to be involved in the regeneration of several organs following tissue damage. In this section, we will review the current understanding of the functions and molecular mechanisms of the Hippo pathway in regulating tissue regeneration.

### Intestine

**Figure 2:**
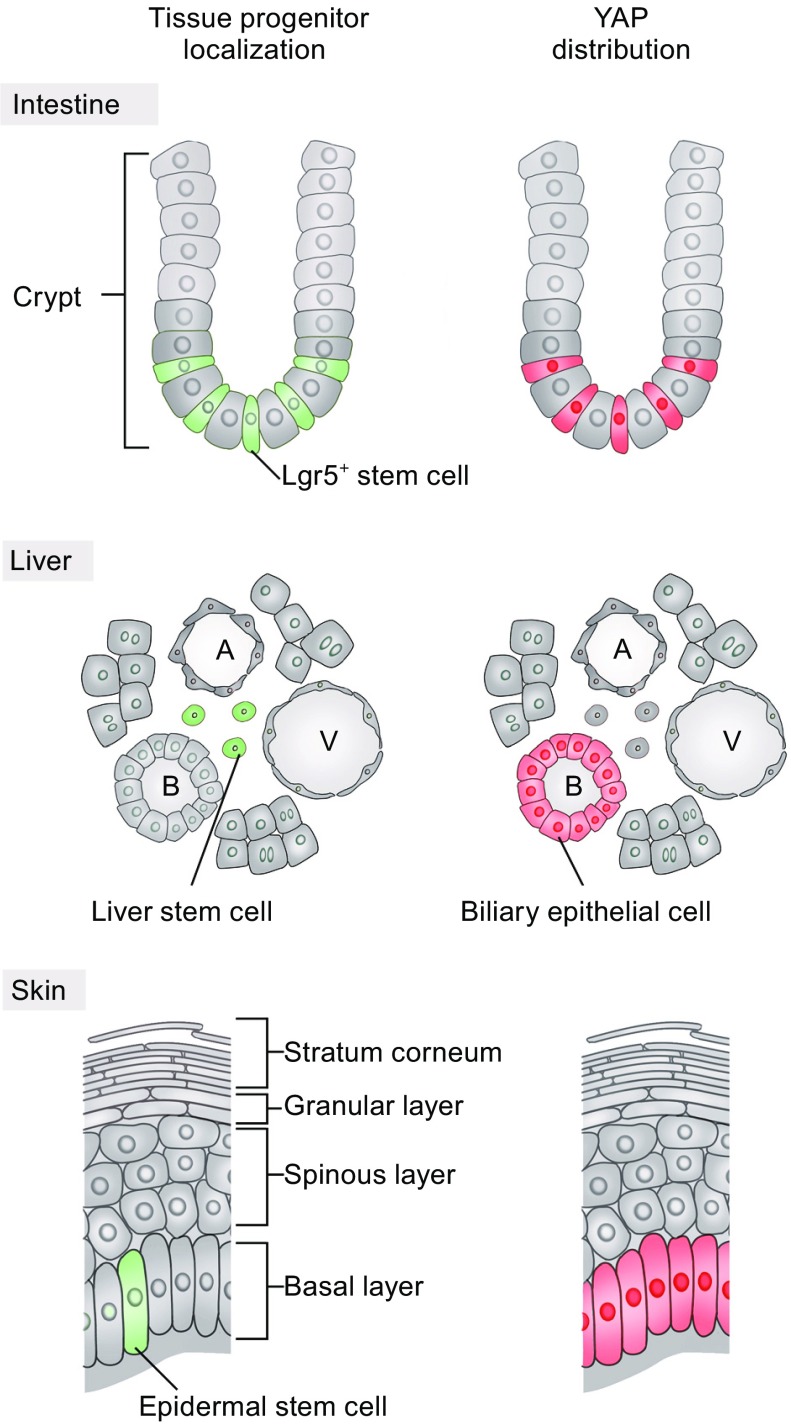
**Localization of tissue resident stem cells and YAP expression in intestine, liver, and skin**. The cellular organization of intestine crypt, portal area of liver, and epidermis are depicted. Cells shaded in green indicate tissue resident stem cells, and red paint indicates YAP expressing. Abbreviations: A, hepatic artery; V, portal vein; B, bile duct

The intestinal epithelium undergoes rapid turnover, and most differentiated cells are replaced with newer ones in less than a week (Barker, 2014). This self-renewal capability is dependent on intestinal stem cells (ISCs)—the crypt base columnar (CBC) cells marked with the leucine-rich repeat-containing GPCR5 (Lgr5) (Fig. 2). Lgr5^+^ ISCs are actively cycling, and a single cell can grow a complete minigut comprised of all types of intestinal epithelial cells, including enterocytes, goblet cells, enteroendocrine, and Paneth cells. A population of quiescent ISCs (stem cells at +4 positions relative to the crypt bottom) may give rise to additional Lgr5^+^ cells in response to tissue damage to promote regeneration (Li and Clevers, 2010).

To maintain stemness, Lgr5^+^ stem cells require niche factors provided by surrounding cells such as nearby Paneth cells and myofibroblasts underneath the epithelial lining, and the function of these niche factors regulate diverse signaling pathways such as Wnt, BMP, and EGF (Crosnier et al., 2006). Wnt signaling is instrumental in intestinal homeostasis, as indicated by the essential role of Wnt3, R-spondin, and downstream β-Catenin/TCF transcription regulators in stem cell maintenance, and Lgr5 is actually a target gene of Wnt signaling (Korinek et al., 1998; Kim et al., 2005; Sato et al., 2011). Recently, the Hippo pathway has also been reported to play an important role in intestinal stem cell self-renewal and regeneration (Yu et al., 2015a). YAP is mainly expressed in Lgr5^+^ stem cells in adult intestine (Barry et al., 2012), suggesting a role for the Hippo pathway in regulating ISC function (Fig. 2). Indeed, overexpression of *Yap*, suppression of *Lats1*/*2*, deletion of *Mst1*/*2*, or deletion of *Sav1* specifically in the intestine all lead to expansion of ISCs and defective cell differentiation, as evidenced by the loss of Paneth cells and goblet cells in the small intestine (Camargo et al., 2007; Lee et al., 2008; Cai et al., 2010; Zhou et al., 2011; Imajo et al., 2014). Surprisingly, mice with conditional knockout of *Yap* and *Taz* exhibit no visible abnormalities (Cai et al., 2010; Zhou et al., 2011; Azzolin et al., 2014). Thus, this suggests that in adult intestine, YAP is not absolutely required for normal tissue homeostasis, and high YAP activity results in a hyperplasia phenotype mainly due to the accumulation of immature cells.

However, the Hippo pathway appears indispensable for intestine tissue regeneration. In mice, tissue injuries such as dextran sodium sulfate (DSS) treatment and gamma radiation represent acute colitis and radiation enteritis, respectively. Following injuries, the intestinal epithelium undergoes an ordered regenerative program. YAP protein levels are dramatically induced following DSS treatment, and YAP is distributed in both cytoplasm and nuclei of all cells in regenerating crypts (Cai et al., 2010). Similarly, YAP is also activated following gamma irradiation, and YAP shows predominant nuclear localization (Gregorieff et al., 2015). In *Yap* cKO or *Yap*/*Taz* dcKO mice following DSS treatment or irradiation, the intestinal epithelium regeneration is defective and loss of crypts is profound (Cai et al., 2010; Gregorieff et al., 2015). *Yap* deficient mice exhibit a dramatic reduction of crypt proliferation, and the ISCs marker Olfm4 is strongly downregulated. Furthermore, transient activation of YAP may reprogram Lgr5^+^ ISCs by partial inhibiting the Wnt pathway, which prevents ISCs from differentiating into Paneth cells and drives a pro-regenerative program through activation of the EGFR signaling pathway (Gregorieff et al., 2015), indicating an essential role for YAP (and TAZ) in intestinal regeneration.

YAP/TAZ have been shown to induce both proliferation of crypt cells and differentiation of ISCs into goblet cells, in which YAP and the TEADs regulate ISCs proliferation, while YAP and Kruppel-like factor 4 (Klf4) regulate goblet cell differentiation (Imajo et al., 2014). However, an inhibitory role for YAP in intestinal regeneration has been observed in another study: overexpression of a constitutively-active *Yap* (S127A mutant) in the mouse intestine leads to the loss of proliferative crypts, and *Yap* knockout results in hyperplastic crypts after whole-body irradiation. In the *Yap* KO intestine, Wnt target genes are upregulated which leads to expansion of ISCs and Paneth cells (Barry et al., 2012). Considering the pivotal role of Wnt signaling in ISCs, the discrepancy between this study and others may be due to the differences in the extent and duration of Wnt inhibition by YAP/TAZ. In addition, intestinal epithelial cells consistently communicate with resident immune cells, and a role of the Hippo pathway in immune response has been revealed recently (Moroishi et al., 2016), thus the differences in mouse immune background and immune-epithelial interaction may also contribute to inconsistent results. Nevertheless, further investigation is required to fully understand the function of YAP/TAZ in intestinal regeneration.

### Liver

The liver has a remarkable regenerative capacity following chemical injury or partial hepatectomy. In response to liver injury, mature hepatocytes proliferate to compensate for cell loss, and tissue resident progenitors also emerge and participate in the regenerative process (Fig. 2). Different populations of cells around the portal area have been suggested as liver progenitor cells, such as bipotent oval cells which give rise to both hepatocytes and cholangiocytes (Miyajima et al., 2014). Recently, the Hippo pathway has been recognized as an essential regulator for regulating liver homeostasis and regeneration.

The Hippo signaling is a crucial regulator in controlling liver development and tumorigenesis. YAP upregulates TGF-β signaling to trigger proliferation of biliary epithelial cells (BEC), and reduces Hnf4α expression to inhibit hepatocyte differentiation (Lee et al., 2016). Liver-specific deletion of *Yap* leads to the loss of biliary epithelial cells, and the liver failed to develop bile ducts (Zhang et al., 2010; Lee et al., 2016). On the other hand, YAP activity is decreased during hepatocyte differentiation, and mature hepatocytes have low YAP expression and nuclear accumulation (Yimlamai et al., 2014; Yi et al., 2016). Conditional activation of YAP leads to liver overgrowth and cancer (Camargo et al., 2007; Dong et al., 2007). Similarly, liver-specific deletion of *Mst1*/*2*, *Lats1*/*2*, *Sav1*, or *Nf2* results in expansion of progenitors, liver enlargement, and liver cancer. The tumor nodules display oval cell accumulation and characteristics of hepatocellular carcinoma (HCC) and cholangiocarcinoma (Zhou et al., 2009; Benhamouche et al., 2010; Lee et al., 2010; Lu et al., 2010; Song et al., 2010; Zhang et al., 2010; Lee et al., 2016; Yi et al., 2016). Surprisingly, Yap^S112A^ knock-in mice are phenotypically normal despite that YAP shows prominent nuclear accumulation (Chen et al., 2015). Further analysis reveals that YAP/TAZ could induce a negative feedback regulation of the Hippo pathway by inducing the expression of LAST1/2 and NF2, which in turn leads to decrease of YAP/TAZ protein level (Chen et al., 2015; Moroishi et al., 2015).

YAP activity also influences liver cell fate during regeneration. In adult liver, YAP is mainly localized to the bile ductal epithelium (Yimlamai et al., 2014) (Fig. 2). Upon liver injury and inflammation, YAP is transiently activated, which promotes proliferation of progenitors and represses hepatocyte differentiation. Deletion of *Yap* in the adult liver causes inhibition of hepatocyte and bile duct proliferation after cholestatic injury (Bai et al., 2012; Su et al., 2015). On the contrary, acute deletion of *Lats1*/*2* in adult mice leads to rapid immature BEC expansion, hepatomegaly, and lethality (Lee et al., 2016). Hepatocyte-specific activation of YAP causes the emergence of cells sharing a similar identity with ductal cells, which is likely due to hepatocyte dedifferentiation as a result of Notch activation (Yimlamai et al., 2014). Taken together, appropriate YAP protein levels are crucial for controlling hepatoblast proliferation and differentiation.

### Skin

The skin is the largest organ that protects the organism from external lesions. The epidermis is continuously renewed to maintain skin homeostasis. Epidermal tissue self-renewal and wound healing are mainly dependent on epidermal stem cells (Solanas and Benitah, 2013; Goodell et al., 2015).

YAP is highly expressed and predominantly nuclear in the early embryonic epidermal progenitors, and is essential for the proliferative capacity of progenitors and the development of the epidermis (Fig. 2). Epidermis-specific deletion of *Yap* at early embryonic stage causes lethality, the skin of these mice is thinner and deficient in epidermal tissue, a notable reduction of both progenitor cells and proliferative basal cells are also observed (Schlegelmilch et al., 2011). On the other hand, the mice carrying a constitutively-active form of YAP (S127A) shows significantly increased proliferation of basal epidermal progenitors, a thicker epidermis, and hyperkeratinization of skin (Schlegelmilch et al., 2011). MST1/2 are activated during keratinocyte differentiation, deletion of their scaffold protein encoded by *Sav1* also causes perinatal lethality and the embryonic epidermal hyperplasia (Lee et al., 2008). Previous work has identified GPCRs as crucial upstream regulators of Hippo signaling (Yu et al., 2012; Yu et al., 2014). Epidermal-specific deletion of *Gnas* leads to expansion of stem cell compartments and basal-cell carcinoma-like lesions due to PKA inactivation and in part, YAP activation (Iglesias-Bartolome et al., 2015).

High YAP activity also affects differentiation of epidermal progenitors and wound healing. Deletion of *Yap* leads to a reduction of cell growth, inhibition of keratinocyte differentiation, and delay in wound healing (Elbediwy et al., 2016). In contrast, terminal differentiation of keratinocytes is blocked in *Yap* (S127A) transgenic mice (Schlegelmilch et al., 2011; Zhang et al., 2011). Thus, these studies demonstrate that YAP activity is crucial to maintain epidermal homeostasis and is indispensable for the wound healing process.

### Heart

Heart growth is strictly restricted and it can be generally divided into two phases: 1) fetal heart growth is mainly due to the proliferation of cardiomyocytes; 2) soon after birth, cardiomyocytes stop proliferating, and heart size is principally controlled by the size of cardiomyocytes. Cardiomyocyte loss is a major pathogenic mechanism leading to heart failure. However, unlike other organs, the heart has been considered as a non-regenerative organ. Recently, some studies have reported that cardiomyocytes also maintain limited regenerative capacity, and cardiac progenitors which may contribute to cardiac regeneration have also been identified at different developmental stages (Laflamme, 2011; Porrello and Olson, 2014; Zhou et al., 2015). Moreover, several studies have shown that the Hippo pathway plays a crucial role in maintaining basal heart homeostasis and regulating cardiomyocyte proliferation and cardiac regeneration. It’s noteworthy that the regenerative potential of adult heart is very low, and the limited recovery of morphology and function of heart following injury may be better referred as tissue repair.

YAP is expressed in the myocardium of both the fetal and postnatal mouse heart (von Gise et al., 2012; Lin et al., 2016). From neonatal to adult stage, the expression of YAP decreases, whereas the expression of VGLL4 (a competitive inhibitor of YAP, Fig. 1) increases gradually, suggesting a decrease in YAP activity with age (Lin et al., 2016). YAP simulates cardiomyocyte proliferation in a TEAD-dependent manner (von Gise et al., 2012; Morikawa et al., 2015; Lin et al., 2016), and YAP is crucial for the development of the embryonic heart, as deletion of *Yap* in the embryonic heart leads to lethality at E10.5 (Xin et al., 2011). Postnatal deletion of *Yap* also leads to increased myocardial fibrosis, cardiomyocyte apoptosis, and decreased cardiomyocyte proliferation, thereby resulting in dilated cardiomyopathy and premature death (Del Re et al., 2013; Xin et al., 2013). Embryonic deletion of *Sav1* leads to the enlargement of the heart and excessive cardiomyocyte proliferation (Heallen et al., 2011). Overexpression of *Lats2* in the mouse heart represses cardiac hypertrophy and reduces ventricle size without influencing myocardial apoptosis (Matsui et al., 2008). Thus, the Hippo pathway is indispensable for regulating embryonic heart development and maintaining basal heart homeostasis.

Recently, several groups have reported that the Hippo pathway plays a role in cardiac regeneration. Activated YAP could reduce myocardia injury and promote cardiac function (Lin et al., 2014). Inhibition of endogenous *Lats2* reduces myocardial apoptosis under stress (Matsui et al., 2008). Overexpression of *Mst1* in mice induces apoptosis and leads to lethal cardiomyopathy (Yamamoto et al., 2003; Delre et al., 2014). Suppression of endogenous *Mst1* prevents cardiomyocyte apoptosis, cardiac dysfunction, and fibrosis in the remodeling heart without influencing cardiomyocyte hypertrophy (Odashima et al., 2007). Cardiac-specific deletion of *Mst2* does not affect cardiomyocyte proliferation in the neonatal or adult heart, but reduces the pathological cardia hypertrophic response under pressure overload (Zi et al., 2014). Loss of *Sav1* in adult mouse cardiomyocytes promotes cell cycle entry and cytokinesis and enhances cardiomyocyte regeneration after myocardia injury (Heallen et al., 2013; Morikawa et al., 2015). Thus, YAP activation represents an attractive approach for promoting heart regeneration.

YAP activity may promote heart regeneration by multiple mechanisms. Expression profiling analysis shows that YAP induces expression of genes related to cell proliferation, DNA synthesis, and cytoskeletal remodeling (von Gise et al., 2012; Morikawa et al., 2015). In addition, YAP stimulates IGF-1 and Akt signaling to reduce cardiomyocyte apoptosis (Xin et al., 2011; Del Re et al., 2013; Xin et al., 2013). Moreover, YAP also binds with different transcriptional factors in the heart, such as FoxO1 and Pitx2, and promotes the expression of genes involved antioxidant response (Shao et al., 2014; Tao et al., 2016).

Together, all these studies demonstrate that appropriate YAP activity is crucial for embryonic heart development and basal heart homeostasis, and YAP could stimulate cardiomyocyte proliferation and cardiac regeneration in response to heart injury such as myocardial ischemia. However, recent studies have principally focused on the effect of the Hippo pathway on cardiomyocytes but not on cardiac fibroblasts or potential cardiac stem cells. Cardiac fibroblasts form one of the largest pools of cells in the heart and contribute to the normal structure and function of the myocardium (Souders et al., 2009). It is reported that cardiac fibroblasts could be directly reprogrammed into adult cardiomyocyte-like cells (Qian et al., 2012). Therefore, it will be important to study the relationship between Hippo pathway and non-myocyte cells in the heart.

### Nervous system

Neural stem cells (NSCs) are capable of self-renewal and generate multiple neuronal and glial lineages, which exist in both the fetal and adult nervous system in mammals. The cell cycle is strictly coordinated in NSCs to ensure precise neurogenesis (Bond et al., 2015). Recent findings indicate a crucial role for Hippo signaling in controlling NSC proliferation, fate determination, differentiation, and maturation.

YAP is selectively expressed in NSCs and astrocytes, but not neurons. In astrocytes, YAP is required for astrocytic proliferation. Deletion of *Yap* in NSCs or astrocyte leads to impaired astrogliogenesis and increased neocortical neurodegeneration (Huang et al., 2016). In the neural tube of the mouse, chicken, and frog, YAP is expressed in the ventricular zone progenitor cells and co-localizes with the neural progenitor cell marker Sox2. YAP activation leads to decreased neuronal differentiation and expansion of the neural progenitor cell population, which in part is due to the upregulation of stemness genes such as cyclin D1 (Cao et al., 2008). On the contrary, repression of either YAP or TEADs in the neural tube causes a significant increase in cell death, cell cycle exit, and differentiation of neuronal cells (Cao et al., 2008). In addition, YAP is necessary for proliferation of ependymal progenitor cells, apical attachment of progenitor cells, and maintaining the integrity of the ventricular lining of the aqueduct. Nervous-specific deletion of *Yap* in the brain obstructs the rostral aqueduct and leads to hydrocephalus (Park et al., 2016). Moreover, YAP/TAZ are also important for the morphogenesis of peripheral nerves, Schwann cells specific knockout of YAP/TAZ in mice leads to reduced cell proliferation, impaired radial sorting, and defective myelination (Poitelon et al., 2016). NF2 is localized in the apical region of NPCs and plays a crucial role in restricting NPC expansion by negatively regulating YAP/TAZ activity. NF2 promotes corpus callosum development and hippocampal morphogenesis, and deletion of *Nf2* in the mouse dorsal telencephalon causes a significant expansion of the NPCs at hippocampus and neocortex, resulting in dysgenesis of the corpus callosum and malformation of the hippocampus (Lavado et al., 2013; Lavado et al., 2014).

## The Hippo Pathway in *Drosophila* Tissue Regeneration

The function of the Hippo pathway in tissue regeneration has also been studied in *Drosophila.* The *Drosophila* midgut is equivalent to the small intestine in mammals. In *Drosophila*, the Hippo pathway is also critical for maintaining midgut homeostasis. The Hippo pathway restricts proliferation of ISCs under normal physiological conditions. Suppression of Wts or Hpo, mutation of the Kibra binding partner Pez, or loss of Msn increases ISC proliferation and causes a significant hyperplasia phenotype in the midgut (Ren et al., 2010; Shaw et al., 2010; Staley and Irvine, 2010; Poernbacher et al., 2012; Li et al., 2014). However, high Yki activity is required for injury-related proliferation of ISCs (Karpowicz et al., 2010; Ren et al., 2010; Staley and Irvine, 2010; Poernbacher et al., 2012). The activation of Yki leads to the production of unpaired (Upd) family cytokines and EGFR ligands, which then activate Jak/Stat and EGFR signaling and accelerate ISC division during intestine regeneration (Karpowicz et al., 2010; Ren et al., 2010; Shaw et al., 2010).

In *Drosophila* nervous system, NSCs remain quiescent at early larval stages and proliferate at embryonic and adult phases, and the Hippo pathway has been shown to control the quiescence of NSCs. Yki is inactive and localized in the cytoplasm when NSCs are quiescent, and Yki will relocate to the nucleus to regulate NSC proliferation and growth during NSC reactivation. Suppression of Hippo pathway upstream regulators such as *Wts* leads to premature exit from quiescence and reactivation of NSCs (Ding et al., 2016). Poon et al. also demonstrated that the Hippo pathway restricts proliferation of neural stem cells, controls neuroblast reactivation from quiescence during postembryonic neurogenesis of *Drosophila*, and perturbation of *Tao*, *Hpo*, or *Wts*, or overexpression of *Yki*, leads to brain overgrowth (Poon et al., 2016). Moreover, the Hippo pathway can modulate asymmetric cell division of NSCs (Keder et al., 2015) and regulate glial cell proliferation (Reddy and Irvine, 2011). In the future, it will be interesting to study the function of the Hippo pathway in neural degenerative diseases or neural regeneration following injuries.

## The Hippo Pathway and Regenerative Medicine

Regenerative medicine refers to medical approaches which promote functional regeneration of damaged tissues or organs, such as stimulation of intrinsic regenerative/repair mechanisms by molecular therapy, or transplantation of tissues or stem/progenitor cells cultured in laboratories (Lane et al., 2014). Due to the shortage of donors compared to the increasing needs of tissue/organ transplantation, there is an urgent need for the development of novel regenerative medicines.

**Figure 3:**
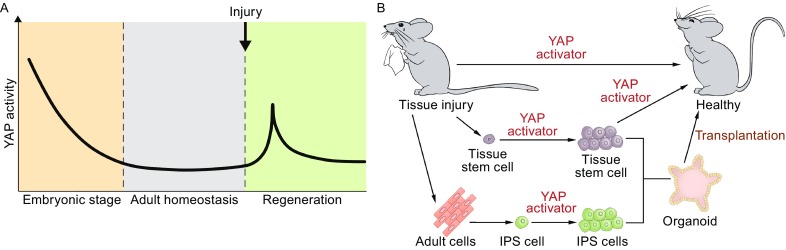
**The Hippo signaling in tissue regeneration and regenerative medicine**. (A) YAP/TAZ activity is declined to a base line level after birth, and immediately reactivated in a transient manner following tissue injuries. (B) The potential of YAP/TAZ activation in regenerative medicine. YAP/TAZ activators or effectors (either small or macro molecules) directly induce tissue regenerative program *in vivo*; in addition, YAP/TAZ activation may promote expansion of tissue resident stem cells or iPSCs *in vitro*, which in turn can be transplanted back to injury sites to facilitate tissue regeneration

YAP/TAZ activity is generally high during embryonic development, but soon declines to a basal level after birth. During tissue injury, YAP/TAZ activity is immediately reactivated in a transient manner, and transient activation of YAP/TAZ can promote expansion of progenitors or dedifferentiation of mature cells to facilitate tissue regeneration (Fig. 3A). Thus, activation of YAP/TAZ is a potential strategy to promote tissue regeneration.

The Hippo pathway consists of a kinase cascade, and therefore, inhibiting upstream kinases represents an ideal approach to activate YAP/TAZ. Systematic or local delivery of Hippo pathway kinase inhibitors could, in principle, induce this regenerative program (Fig. 3B). Recently, an MST1/2 inhibitor has been discovered and has shown good efficacy in promoting liver and intestinal regeneration (Fan et al., 2016). Inhibitors for MAP4Ks or LATS1/2 may have a similar effect in promoting regeneration. Gene therapy is an effective approach in regenerative medicine (Ruiz and Regueiro, 2012), introducing small interfering RNA or microRNA mimics targeting the pathway components or YAP target genes by viral- or viral free- approaches may benefit tissue regeneration (Yin et al., 2014).

An alternative approach is to deliver macromolecules to damaged tissues to facilitate tissue regeneration. Some YAP/TAZ targeting genes encode secretory proteins, and these proteins may have regenerative potential. Indeed, YAP targets Epiregulin and CTGF have been shown to promote tissue repair in the mouse intestine and zebrafish spinal cord, respectively (Gregorieff et al., 2015; MH et al., 2016).

In addition to *in vivo* reprogramming of regenerative process using a molecular approach, regeneration may also be promoted by transplantation of *in vitro* expanded progenitors, organoids, or tissues (Fig. 3B). In recent years, a variety of organoids have been cultured successfully *in vitro*, including the stomach, liver, kidney, lung, gut, brain, and retina (Clevers, 2016). However, it is still difficult to control the complicated biological parameters such as the cell type, organization, and cell-cell or cell-matrix interactions within an organoid system (Yin et al., 2016). In a recent study, intestinal organoid formation has been fine-tuned by differential YAP activity associated with matrix stiffness (Gjorevski et al., 2016). Moreover, YAP/TAZ transient activation can efficiently convert differentiated mammary, neuronal, and pancreatic cells into a progenitor cell state, and these cells can form organoids and be used for transplantation (Panciera et al., 2016). Thus, modulating the Hippo pathway may represent a useful approach for enrichment of progenitor cells or differentiated organoids for regenerative medicine.

Given the importance of the Hippo pathway in cell plasticity, novel and specific activators of YAP/TAZ may be a powerful tool for promoting tissue regeneration. While the Hippo field is largely focused on developing YAP/TAZ inhibitors for treating cancer (Gong and Yu, 2015), it might be equally important to develop YAP/TAZ activators for regenerative medicine. Moreover, long term activation of YAP/TAZ may lead to tumorigenesis, thus caution should be taken when using YAP/TAZ activators in regenerative medicine.

## References

[CR1] Aylon Y, Sarver A, Tovy A, Ainbinder E, Oren M (2014). Lats2 is critical for the pluripotency and proper differentiation of stem cells. Cell Death Differ.

[CR2] Azzolin L, Panciera T, Soligo S, Enzo E, Bicciato S, Dupont S, Bresolin S, Frasson C, Basso G, Guzzardo V (2014). YAP/TAZ incorporation in the β-catenin destruction complex orchestrates the Wnt response. Cell.

[CR3] Bai H, Zhang N, Xu Y, Chen Q, Khan M, Potter JJ, Nayar SK, Cornish T, Alpini G, Bronk S (2012). Yes-associated protein regulates the hepatic response after bile duct ligation. Hepatology.

[CR4] Barker N (2014). Adult intestinal stem cells: critical drivers of epithelial homeostasis and regeneration. Nat Rev Mol Cell Bio.

[CR5] Barry ER, Morikawa T, Butler BL, Shrestha K, de la Rosa R, Yan KS, Fuchs CS, Magness ST, Smits R, Ogino S (2012). Restriction of intestinal stem cell expansion and the regenerative response by YAP. Nature.

[CR6] Benhamouche S, Curto M, Saotome I, Gladden AB, Liu CH, Giovannini M, McClatchey AI (2010). Nf2/Merlin controls progenitor homeostasis and tumorigenesis in the liver. Genes Dev.

[CR7] Beyer TA, Weiss A, Khomchuk Y, Huang K, Ogunjimi AA, Varelas X, Wrana JL (2013). Switch enhancers interpret TGF-β and Hippo signaling to control cell fate in human embryonic stem cells. Cell Rep.

[CR8] Bond AM, Ming G, Song H (2015). Adult mammalian neural stem cells and neurogenesis: five decades later. Cell Stem Cell.

[CR9] Borowiak M, Wigler MH (2004). Met provides essential signals for liver regeneration. Proc Natl Acad Sci USA.

[CR10] Cai J, Zhang N, Zheng Y, de Wilde RF, Maitra A, Pan D (2010). The Hippo signaling pathway restricts the oncogenic potential of an intestinal regeneration program. Genes Dev.

[CR11] Camargo FD, Gokhale S, Johnnidis JB, Fu D, Bell GW, Jaenisch R, Brummelkamp TR (2007). YAP1 increases organ size and expands undifferentiated progenitor cells. Curr Biol.

[CR12] Cao X, Pfaff SL, Gage FH (2008). YAP regulates neural progenitor cell number via the TEA domain transcription factor. Genes Dev.

[CR13] Carlson BM (2007). Principles of regenerative biology.

[CR14] Chen Q, Zhang N, Xie R, Wang W, Cai J, Choi KS, David KK, Huang B, Yabuta N, Nojima H (2015). Homeostatic control of Hippo signaling activity revealed by an endogenous activating mutation in YAP. Genes Dev.

[CR15] Chung H, Lee BK, Uprety N, Shen W, Lee J, Kim J (2016). Yap1 is dispensable for self-renewal but required for proper differentiation of mouse embryonic stem (ES) cells. Embo Rep.

[CR16] Clevers H (2016). Modeling development and disease with organoids. Cell.

[CR17] Cockburn K, Biechele S, Garner J, Rossant J (2013). The hippo pathway member Nf2 Is required for inner cell mass specification. Curr Biol.

[CR18] Crosnier C, Stamataki D, Lewis J (2006). Organizing cell renewal in the intestine: stem cells, signals and combinatorial control. Nat Rev Genet.

[CR19] Del Re DP, Yang Y, Nakano N, Cho J, Zhai P, Yamamoto T, Zhang N, Yabuta N, Nojima H, Pan D (2013). Yes-associated protein isoform 1 (Yap1) promotes cardiomyocyte survival and growth to protect against myocardial ischemic injury. J Biol Chem.

[CR20] Delre D, Matsuda T, Zhai P, Maejima Y, Jain MR, Liu T, Li H, Hsu CP, Sadoshima J (2014). Mst1 promotes cardiac myocyte apoptosis through phosphorylation and inhibition of Bcl-xL. Mol Cell.

[CR21] Ding R, Weynans K, Bossing T, Barros CS, Berger C (2016). The Hippo signalling pathway maintains quiescence in Drosophila neural stem cells. Nat Commun.

[CR22] Dong J, Feldmann G, Huang J, Wu S, Zhang N, Comerford SA, Gayyed MF, Anders RA, Maitra A, Pan D (2007). Elucidation of a universal size-control mechanism in drosophila and mammals. Cell.

[CR23] Elbediwy A, Vincent-Mistiaen ZI, Spencer-Dene B, Stone RK, Boeing S, Wculek SK, Cordero J, Tan EH, Ridgway R, Brunton VG (2016). Integrin signalling regulates YAP and TAZ to control skin homeostasis. Development.

[CR24] Fan F, He Z, Kong LL, Chen Q, Yuan Q, Zhang S, Ye J, Liu H, Sun X, Geng J (2016). Pharmacological targeting of kinases MST1 and MST2 augments tissue repair and regeneration. Sci Transl Med.

[CR25] Gjorevski N, Sachs N, Manfrin A, Giger S, Bragina ME, Ordóñez-Morán P, Clevers H, Lutolf MP (2016). Designer matrices for intestinal stem cell and organoid culture. Nature.

[CR26] Gong R, Yu FX (2015). Targeting the Hippo pathway for anti-cancer therapies. Curr Med Chem.

[CR27] Goodell MA, Nguyen H, Shroyer N (2015). Somatic stem cell heterogeneity: diversity in the blood, skin and intestinal stem cell compartments. Nat Rev Mol Cell Biol.

[CR28] Gregorieff A, Liu Y, Inanlou MR, Khomchuk Y, Wrana JL (2015). Yap-dependent reprogramming of Lgr5+ stem cells drives intestinal regeneration and cancer. Nature.

[CR29] Gurtner GC, Sabine W, Yann B, Longaker MT (2012). Wound repair and regeneration. Nature.

[CR30] Halder G, Johnson RL (2011). Hippo signaling: growth control and beyond. Development.

[CR31] Han M, Yang X, Farrington JE, Muneoka K (2003). Digit regeneration is regulated by Msx1 and BMP4 in fetal mice. Development.

[CR32] Heallen T, Zhang M, Wang J, Bonilla-Claudio M, Klysik E, Johnson RL, Martin JF (2011). Hippo pathway inhibits Wnt signaling to restrain cardiomyocyte proliferation and heart size. Science.

[CR33] Heallen T, Morikawa Y, Leach J, Tao G, Willerson JT, Johnson RL, Martin JF (2013). Hippo signaling impedes adult heart regeneration. Development.

[CR34] Hirate Y, Hirahara S, Inoue K, Suzuki A, Alarcon VB, Akimoto K, Hirai T, Hara T, Adachi M, Chida K (2013). Polarity-dependent distribution of angiomotin localizes Hippo signaling in preimplantation embryos. Curr Biol.

[CR35] Huang Z, Hu J, Pan J, Wang Y, Hu G, Zhou J, Mei L, Xiong W (2016). YAP stabilizes SMAD1 and promotes BMP2-induced neocortical astrocytic differentiation. Development.

[CR36] Huh CG, Factor VM, Sánchez A, Uchida K, Conner EA, Thorgeirsson SS (2004). Hepatocyte growth factor/c-met signaling pathway is required for efficient liver regeneration and repair. Proc Natl Acad Sci USA.

[CR37] Iglesias-Bartolome R, Torres D, Marone R, Feng X, Martin D, Simaan M, Chen M, Weinstein LS, Taylor SS, Molinolo AA (2015). Inactivation of a Gαs–PKA tumour suppressor pathway in skin stem cells initiates basal-cell carcinogenesis. Nat Cell Biol.

[CR38] Imajo M, Ebisuya M, Nishida E (2014). Dual role of YAP and TAZ in renewal of the intestinal epithelium. Nat Cell Biol.

[CR39] Karpowicz P, Perez J, Perrimon N (2010). The Hippo tumor suppressor pathway regulates intestinal stem cell regeneration. Development.

[CR40] Keder A, Rives-Quinto N, Aerne BL, Franco M, Tapon N, Carmena A (2015). The Hippo pathway core cassette regulates asymmetric cell division. Curr Biol.

[CR41] Kim KA, Kakitani M, Zhao J, Oshima T, Tang T, Binnerts M, Liu Y, Boyle B, Park E, Emtage P (2005). Mitogenic influence of human R-spondin1 on the intestinal epithelium. Science.

[CR42] Korinek V, Barker N, Moerer P, Van DE, Huls G, Peters PJ, Clevers H (1998). Depletion of epithelial stem-cell compartments in the small intestine of mice lacking Tcf-4. Nat Genet.

[CR43] Laflamme MA (2011). Heart regeneration. Nature.

[CR44] Lane SW, Williams DA, Watt FM (2014). Modulating the stem cell niche for tissue regeneration. Nat Biotechnol.

[CR45] Lavado A, He Y, Pare J, Neale G, Olson EN, Giovannini M, Cao X (2013). Tumor suppressor Nf2 limits expansion of the neural progenitor pool by inhibiting Yap/Taz transcriptional coactivators. Development.

[CR46] Lavado A, Ware M, Pare J, Cao X (2014). The tumor suppressor Nf2 regulates corpus callosum development by inhibiting the transcriptional coactivator Yap. Development.

[CR47] Lee JH, Kim TS, Yang TH, Koo BK, Oh SP, Lee KP, Oh HJ, Lee SH, Kong YY, Kim JM (2008). A crucial role of WW45 in developing epithelial tissues in the mouse. Embo J.

[CR48] Lee KP, Lee JH, Kim TS, Kim TH, Park HD, Byun JS, Kim MC, Jeong WI, Calvisi DF, Kim JM (2010). The Hippo-Salvador pathway restrains hepatic oval cell proliferation, liver size, and liver tumorigenesis. Proc Natl Acad Sci USA.

[CR49] Lee D, Park JO, Kim T, Kim S, Kim T, Kim M, Park GS, Kim J, Kuninaka S, Olson EN (2016). LATS-YAP/TAZ controls lineage specification by regulating TGFβ signaling and Hnf4α expression during liver development. Nat Commun.

[CR50] Li L, Clevers H (2010). Coexistence of quiescent and active adult stem cells in mammals. Science.

[CR51] Li P, Chen Y, Mak KK, Wong CK, Wang CC, Yuan P (2013). Functional role of Mst1/Mst2 in embryonic stem cell differentiation. PLoS ONE.

[CR52] Li Q, Li S, Mana-Capelli S, Roth FR, Danai LV, Amcheslavsky A, Nie Y, Kaneko S, Yao X, Chen X (2014). The conserved misshapen-warts-Yorkie pathway acts in enteroblasts to regulate intestinal stem cells in *Drosophila*. Dev Cell.

[CR53] Lian I, Kim J, Okazawa H, Zhao J, Zhao B, Yu J, Chinnaiyan A, Israel MA, Goldstein LS, Abujarour R (2010). The role of YAP transcription coactivator in regulating stem cell self-renewal and differentiation. Genes Dev.

[CR54] Lin Z, von Gise A, Zhou P, Gu F, Ma Q, Jiang J, Yau AL, Buck JN, Gouin KA, van Gorp PRR (2014). Cardiac-specific YAP activation improves cardiac function and survival in an experimental murine MI model. Circ Res.

[CR55] Lin Z, Guo H, Cao Y, Zohrabian S, Zhou P, Ma Q, VanDusen N, Guo Y, Zhang J, Stevens SM (2016). Acetylation of VGLL4 regulates Hippo-YAP signaling and postnatal cardiac growth. Dev Cell.

[CR56] Lorthongpanich C, Messerschmidt DM, Chan SW, Hong W, Knowles BB, Solter D (2013). Temporal reduction of LATS kinases in the early preimplantation embryo prevents ICM lineage differentiation. Genes Dev.

[CR57] Lu L, Li Y, Kim SM, Bossuyt W, Liu P, Qiu Q, Wang Y, Halder G, Finegold MJ, Lee JS (2010). Hippo signaling is a potent in vivo growth and tumor suppressor pathway in the mammalian liver. Proc Natl Acad Sci USA.

[CR58] Matsui Y, Nakano N, Shao D, Gao S, Luo W, Hong C, Zhai P, Holle E, Yu X, Yabuta N (2008). Lats2 Is a negative regulator of myocyte size in the heart. Circ Res.

[CR59] Miyajima A, Tanaka M, Itoh T (2014). Stem/progenitor cells in liver development, homeostasis, regeneration, and reprogramming. Cell Stem Cell.

[CR60] Mokalled MH, Patra C, Dickson AL, Endo T, Stainier DY, Poss KD (2016). Injury-induced ctgfa directs glial bridging and spinal cord regeneration in zebrafish. Science.

[CR61] Morikawa Y, Zhang M, Heallen T, Leach J, Tao G, Xiao Y, Bai Y, Li W, Willerson JT, Martin JF (2015). Actin cytoskeletal remodeling with protrusion formation is essential for heart regeneration in Hippo-deficient mice. Sci Signal.

[CR62] Moroishi T, Park HW, Qin B, Chen Q, Meng Z, Plouffe SW, Taniguchi K, Yu FX, Karin M, Pan D (2015). A YAP/TAZ-induced feedback mechanism regulates Hippo pathway homeostasis. Genes Dev.

[CR63] Moroishi T, Hayashi T, Pan WW, Fujita Y, Holt MV, Qin J, Carson DA, Guan KL (2016). The Hippo pathway kinases LATS1/2 suppress cancer immunity. Cell.

[CR64] Odashima M, Usui S, Takagi H, Hong C, Liu J, Yokota M, Sadoshima J (2007). Inhibition of endogenous Mst1 prevents apoptosis and cardiac dysfunction without affecting cardiac hypertrophy after myocardial infarction. Circ Res.

[CR65] Pan D (2010). The Hippo signaling pathway in development and cancer. Dev Cell.

[CR66] Panciera T, Azzolin L, Fujimura A, Di Biagio D, Frasson C, Bresolin S, Soligo S, Basso G, Bicciato S, Rosato A (2016). Induction of expandable tissue-specific stem/progenitor cells through transient expression of YAP/TAZ. Cell Stem Cell.

[CR67] Park R, Moon UY, Park JY, Hughes LJ, Johnson RL, Cho S, Kim S (2016). Yap is required for ependymal integrity and is suppressed in LPA-induced hydrocephalus. Nat Commun.

[CR68] Poernbacher I, Baumgartner R, Marada SK, Edwards K, Stocker H (2012). Drosophila Pez acts in Hippo signaling to restrict intestinal stem cell proliferation. Curr Biol.

[CR69] Poitelon Y, Lopez-Anido C, Catignas K, Berti C, Palmisano M, Williamson C, Ameroso D, Abiko K, Hwang Y, Gregorieff A (2016). YAP and TAZ control peripheral myelination and the expression of laminin receptors in Schwann cells. Nat Neurosci.

[CR70] Poon CLC, Mitchell KA, Kondo S, Cheng LY, Harvey KF (2016). The Hippo pathway regulates neuroblasts and brain size in *Drosophila melanogaster*. Curr Biol.

[CR71] Porrello ER, Olson EN (2014). A neonatal blueprint for cardiac regeneration. Stem Cell Res.

[CR72] Qian L, Huang Y, Spencer CI, Foley A, Vedantham V, Liu L, Conway SJ, Fu JD, Srivastava D (2012). In vivo reprogramming of murine cardiac fibroblasts into induced cardiomyocytes. Nature.

[CR73] Qin H, Blaschke K, Wei G, Ohi Y, Blouin L, Qi Z, Yu J, Yeh RF, Hebrok M, Ramalho-Santos M (2012). Transcriptional analysis of pluripotency reveals the Hippo pathway as a barrier to reprogramming. Hum Mol Genet.

[CR74] Qin H, Hejna M, Liu Y, Percharde M, Wossidlo M, Blouin L, Durruthy-Durruthy J, Wong P, Qi Z, Yu J (2016). YAP induces human naive pluripotency. Cell Rep.

[CR75] Reddy BVVG, Irvine KD (2011). Regulation of *Drosophila* glial cell proliferation by Merlin–Hippo signaling. Development.

[CR76] Ren F, Wang B, Yue T, Yun EY, Ip YT, Jiang J (2010). Hippo signaling regulates Drosophila intestine stem cell proliferation through multiple pathways. Proc Natl Acad Sci USA.

[CR77] Ruiz MM, Regueiro JR (2012). New tools in regenerative medicine: gene therapy.

[CR78] Sasaki H (2015). Position- and polarity-dependent Hippo signaling regulates cell fates in preimplantation mouse embryos. Semin Cell Dev Biol.

[CR79] Sato T, van Es JH, Snippert HJ, Stange DE, Vries RG, Van DBM, Barker N, Shroyer NF, Van DWM, Clevers H (2011). Paneth cells constitute the niche for Lgr5 stem cells in intestinal crypts. Nature.

[CR80] Schlegelmilch K, Mohseni M, Kirak O, Pruszak J, Rodriguez JR, Zhou D, Kreger BT, Vasioukhin V, Avruch J, Brummelkamp TR (2011). Yap1 acts downstream of alpha-catenin to control epidermal proliferation. Cell.

[CR81] Shao D, Zhai P, Del Re DP, Sciarretta S, Yabuta N, Nojima H, Lim D, Pan D, Sadoshima J (2014). A functional interaction between Hippo-YAP signalling and FoxO1 mediates the oxidative stress response. Nat Commun.

[CR82] Shaw RL, Kohlmaier A, Polesello C, Veelken C, Edgar BA, Tapon N (2010). The Hippo pathway regulates intestinal stem cell proliferation during Drosophila adult midgut regeneration. Development.

[CR83] Solanas G, Benitah SA (2013). Regenerating the skin: a task for the heterogeneous stem cell pool and surrounding niche. Nat Rev Mol Cell Biol.

[CR84] Song H, Mak KK, Topol L, Yun K, Hu J, Garrett L, Chen Y, Park O, Chang J, Simpson RM (2010). Mammalian Mst1 and Mst2 kinases play essential roles in organ size control and tumor suppression. Proc Natl Acad Sci.

[CR85] Souders CA, Bowers SLK, Baudino TA (2009). Cardiac fibroblast: the renaissance cell. Circ Res.

[CR86] Staley BK, Irvine KD (2010). Warts and Yorkie mediate intestinal regeneration by influencing stem cell proliferation. Curr Biol.

[CR87] Stoick-Cooper CL, Moon RT, Weidinger G (2007). Advances in signaling in vertebrate regeneration as a prelude to regenerative medicine. Genes Dev.

[CR88] Su T, Bondar T, Zhou X, Zhang C, He H, Medzhitov R (2015). Two-signal requirement for growth-promoting function of Yap in hepatocytes. eLife.

[CR89] Tamm C, Böwer N, Annerén C (2011). Regulation of mouse embryonic stem cell self-renewal by a Yes-YAP-TEAD2 signaling pathway downstream of LIF. J Cell Sci.

[CR90] Tao G, Kahr PC, Morikawa Y, Zhang M, Rahmani M, Heallen TR, Li L, Sun Z, Olson EN, Amendt BA (2016). Pitx2 promotes heart repair by activating the antioxidant response after cardiac injury. Nature.

[CR91] Varelas X, Sakuma R, Samavarchi-Tehrani P, Peerani R, Rao BM, Dembowy J, Yaffe MB, Zandstra PW, Wrana JL (2008). TAZ controls Smad nucleocytoplasmic shuttling and regulates human embryonic stem-cell self-renewal. Nat Cell Biol.

[CR92] von Gise A, Lin Z, Schlegelmilch K, Honor LB, Pan GM, Buck JN, Ma Q, Ishiwata T, Zhou B, Camargo FD (2012). YAP1, the nuclear target of Hippo signaling, stimulates heart growth through cardiomyocyte proliferation but not hypertrophy. Proc Natl Acad Sci USA.

[CR93] Whyte JL, Smith AA, Helms JA (2012). Wnt Signaling and Injury Repair. Cold Spring Harb Perspect Biol.

[CR94] Xin M, Kim Y, Sutherland LB, Qi X, Mcanally J, Schwartz RJ, Richardson JA, Basselduby R, Olson EN (2011). Regulation of insulin-like growth factor signaling by Yap governs cardiomyocyte proliferation and embryonic heart size. Sci Signal.

[CR95] Xin M, Kim Y, Sutherland LB, Murakami M, Qi X, McAnally J, Porrello ER, Mahmoud AI, Tan W, Shelton JM (2013). Hippo pathway effector Yap promotes cardiac regeneration. Proc Natl Acad Sci USA.

[CR96] Yamamoto S, Yang G, Zablocki D, Liu J, Hong C, Kim S, Soler S, Odashima M, Thaisz J, Yehia G (2003). Activation of Mst1 causes dilated cardiomyopathy by stimulating apoptosis without compensatory ventricular myocyte hypertrophy. J Clin Invest.

[CR97] Yi J, Lu L, Yanger K, Wang W, Sohn BH, Stanger BZ, Zhang M, Martin JF, Ajani JA, Chen J (2016). Large tumor suppressor homologs 1 and 2 regulate mouse liver progenitor cell proliferation and maturation through antagonism of the coactivators YAP and TAZ. Hepatology.

[CR98] Yimlamai D, Christodoulou C, Galli GG, Yanger K, Pepe-Mooney B, Gurung B, Shrestha K, Cahan P, Stanger BZ, Camargo FD (2014). Hippo pathway activity influences liver cell fate. Cell.

[CR99] Yin H, Kanasty RL, Eltoukhy AA, Vegas AJ, Dorkin JR, Anderson DG (2014). Non-viral vectors for gene-based therapy. Nat Rev Genet.

[CR100] Yin X, Mead BE, Safaee H, Langer R, Karp JM, Levy O (2016). Engineering stem cell organoids. Cell Stem Cell.

[CR101] Yu FX, Guan KL (2013). The Hippo pathway: regulators and regulations. Genes Dev.

[CR102] Yu FX, Zhao B, Panupinthu N, Jewell JL, Lian I, Wang LH, Zhao J, Yuan H, Tumaneng K, Li H (2012). Regulation of the Hippo-YAP pathway by G-protein-coupled receptor signaling. Cell.

[CR103] Yu FX, Luo J, Mo JS, Liu G, Kim YC, Meng Z, Zhao L, Peyman G, Ouyang H, Jiang W (2014). Mutant Gq/11 promote uveal melanoma tumorigenesis by activating YAP. Cancer Cell.

[CR104] Yu F, Meng Z, Plouffe SW, Guan K (2015). Hippo pathway regulation of gastrointestinal tissues. Annu Rev Physiol.

[CR105] Yu FX, Zhao B, Guan KL (2015). Hippo pathway in organ size control, tissue homeostasis, and cancer. Cell.

[CR106] Zhang N, Bai H, David KK, Dong J, Zheng Y, Cai J, Giovannini M, Liu P, Anders RA, Pan D (2010). The Merlin/NF2 tumor suppressor functions through the YAP oncoprotein to regulate tissue homeostasis in mammals. Dev Cell.

[CR107] Zhang H, Pasolli HA, Fuchs E (2011). Yes-associated protein (YAP) transcriptional coactivator functions in balancing growth and differentiation in skin. Proc Natl Acad Sci USA.

[CR108] Zhou D, Conrad C, Xia F, Park JS, Payer B (2009). Mst1 and Mst2 maintain hepatocyte quiescence and suppress hepatocellular carcinoma development through inactivation of the Yap1 oncogene. Cancer Cell.

[CR109] Zhou D, Zhang Y, Wu H, Barry E, Yin Y, Lawrence E, Dawson D, Willis JE, Markowitz SD, Camargo FD (2011). Mst1 and Mst2 protein kinases restrain intestinal stem cell proliferation and colonic tumorigenesis by inhibition of Yes-associated protein (Yap) overabundance. Proc Natl Acad Sci USA.

[CR110] Zhou Q, Li L, Zhao B, Guan KL (2015). The Hippo pathway in heart development, regeneration, and diseases. Circ Res.

[CR111] Zi M, Maqsood A, Prehar S, Mohamed TMA, Abou-Leisa R, Robertson A, Cartwright EJ, Ray SG, Oh S, Lim DS (2014). The mammalian Ste20-like kinase 2 (Mst2) modulates stress-induced cardiac hypertrophy. J Biol Chem.

